# A phenotypic model recapitulating the neuropathology of Parkinson's disease

**DOI:** 10.1002/brb3.138

**Published:** 2013-04-17

**Authors:** Craig F Ferris, Mathieu Marella, Brian Smerkers, Thomas M Barchet, Benjamin Gershman, Akemi Matsuno-Yagi, Takao Yagi

**Affiliations:** 1 Center for Translational NeuroImaging, Northeastern UniversityBoston, Massachusetts; 2 Division of Biochemistry, Department of Molecular and Experimental Medicine, The Scripps Research InstituteLa Jolla, California; 3 State University of New York Upstate Medical UniversitySyracuse, New York; 4 InviCRO, LLCBoston, Massachusetts

**Keywords:** DNA oxidation, dopamine transporter, Lewy bodies, oxidative stress, rotenone, tyrosine hydroxylase, vesicular monoamine transporter

## Abstract

This study was undertaken to develop a phenotypic model recapitulating the neuropathology of Parkinson's disease (PD). Such a model would show loss of dopamine in the basal ganglia, appearance of Lewy bodies, and the early stages of motor dysfunction. The model was developed by subcutaneously injecting biodegradable microspheres of rotenone, a complex I inhibitor in 8–9 month old, ovariectomized Long–Evans rats. Animals were observed for changes in body weight and motor activity. At the end of 11–12 weeks animals were euthanized and the brains examined for histopathological changes. Rotenone treated animals gain weight and appear normal and healthy as compared to controls but showed modest hypokinesia around 5–6 weeks posttreatment. Animals showed loss of dopaminergic (DA) neurons and the appearance of putative Lewy bodies in the substantia nigra. Neuroinflammation and oxidative stress were evidenced by the appearance of activated microglia, iron precipitates, and 8-oxo-2′-deoxyguanosine a major product of DNA oxidation. The dorsal striatum, the projection site of midbrain DA neurons, showed a significant reduction in tyrosine hydroxylase immunostaining, together with an increase in reactive astrocytes, an early sign of DA nerve terminal damage. Levels of vesicular monoamine transporter 2 (VMAT2) were significantly reduced in the dorsal striatum; however, there was an unexpected increase in dopamine transporter (DAT) levels. Old, ovariectomized females treated with rotenone microspheres present with normal weight gain and good health but a modest hypokinesia. Accompanying this behavioral phenotype are a constellation of neuropathologies characteristic of PD that include loss of DA neurons, microglia activation, oxidative damage to nuclear DNA, iron deposition, and appearance of putative Lewy bodies. This phenotypic model recapitulating the neuropathology of Parkinson's disease could provide insight into early mechanisms of pathogenesis and could aid in the identification of biomarkers to identify patients in early stage, PD.

## Introduction

Parkinson's disease (PD) is the second most common neurodegenerative disorder after Alzheimer's and affects nearly 1 in 1000 people globally (de Lau and Breteler 2006). As an age-related disease it affects 1% of the population over the age of 65 years. Although PD is a multisystem disease, the dopaminergic (DA) cells localized in the substantia nigra pars compacta region are mostly affected. These DA neurons have a significant afferent connection to the striatum and form the nigrostriatal dopaminergic system which is critical in motor, cognitive, and limbic function. Central to the neuropathogenesis of PD is the unwanted aggregation of α-synuclein protein. The pathology of synuclein aggregation to form Lewy neurites and Lewy bodies takes several years to develop, ultimately associated with the destruction of DA neurons in the SN and the cardinal symptoms of PD – resting tremor, rigidity, bradykinesia, and postural instability. Unfortunately these symptoms reflect the functional loss of the DA system from which there is no known treatment for recovery. Efforts to treat PD have focused on symptomatic relief in late stages of the disease with no success. There is no therapy today that can alter the course or delay the progression of the disease. When a patient becomes symptomatic, majority of the DA neurons and neurotransmitters are irreparably lost. Therefore, identifying subjects at risk for PD while they are presymptomatic would help in developing early intervention strategies, which might arrest disease progression and possibly restore neuronal function.

This study was undertaken to develop an animal model of PD that recapitulates disease progression in humans. Such a model would provide insight into early mechanisms of pathogenesis providing greater latitude in the development of new interventions and means of testing new therapeutics. Moreover, such a model could aid in the identification of biomarkers that translate to the clinic in the effort to identify patients in early stage, presymptomatic PD. While the etiology of idiopathic PD is not known, there is an ever increasing body of literature documenting changes in the biochemistry and cell biology of the nigrostrial dopaminergic pathway in animal models that have corroborated findings in human studies. These changes include disruption of mitochondrial respiration, microglia activation, neuroinflammation, oxidative stress, and misfolding and aggregation of α-synuclein protein. Indeed, the definitive diagnosis of iodiopathic PD is only made after neuropathological examination to identify the presence of α-synuclein immunostaining in Lewy neurites and Lewy bodies (Mikolaenko et al. 2005; Litvan et al. 2007). The presence of α-synuclein inclusions in neurons is the hallmark of presymptomatic and symptomatic PD (Braak et al. 1995; Trojanowski et al. 1998; Gwinn-Hardy 2002; Thal et al. 2004; Dickson et al. 2008).

The model we present uses the mitochondrial toxin rotenone. The seminal paper by Greenamyre's laboratory reported chronic, systemic exposure to the pesticide rotenone reproduces features of PD in rats (Betarbet et al. 2000). Motor dysfunction, loss of DA in the nigrostrial system, modest degeneration of noradrenergic neurons of the locus ceruleus, and development of α-synuclein aggregates and Lewy bodies-like inclusions all occur with rotenone treatment. The work done by Greenamyre and Sherer with rotenone gave the promise of a very favorable animal model to study the mitochondrial dysfunction, synucleinopathy, microglia activation, and oxidative stress associated with the etiopathogenesis of PD (Sherer et al. 2002, 2003a,b,c; Testa et al. 2005; Betarbet et al. 2006). Unfortunately, animal morbidity combined with high experimental variability and a low incidence of fulminating PD diminished enthusiasm for the model (Fleming et al. 2004; Lapointe et al. 2004; Zhu et al. 2004; Phinney et al. 2006). However, Yagi and colleagues at The Scripps Research Institute reported a method in rats for releasing rotenone through subcutaneous, biodegradable microspheres (Marella et al. 2008) that provides a gradual increase in plasma rotenone over the first few weeks followed by slow and steady decline over the subsequent months. With some modifications to their original methods we produced a constellation of neuropathologies characteristic of PD that include loss of DA neurons, microglia activation, oxidative damage to nuclear DNA, iron deposition, and appearance of putative Lewy bodies. We consider this to be a phenotypic animal model recapitulating the neuropathology of human PD.

## Methods

### Animals

Adult, female Long–Evan rats were purchased from Harlan Sprague Dawley, Inc (Indianapolis, IN). Animals were housed in Plexiglas cages (two per cage) and maintained in ambient temperature (22–24°C) on a 12:12 light:dark cycle (lights on at 0900 h). Food and water were provided ad libitum. All animals were acquired and cared for in accordance with the guidelines published in the NIH Guide for the Care and Use of Laboratory Animals. All methods and procedures described below were preapproved by the Northeastern University Institutional Animal Care and Use Committee (NU-IACUC).

The present rotenone model using biodegradable microspheres for toxin delivery was taken from Marella and coworkers (2008). These researchers improved on an earlier rotenone microsphere model published by Huang et al. (2006), by working with older, 5-month-aged, male rats. Indeed, most published data using rats to model PD come from young adults animal, 2–3 months of age. It was our intention to use this model to follow disease progression with noninvasive magnetic resonance imaging and mole-cular imaging using single-photon emission computed tomography (SPECT). The behavior and imaging studies were performed at the Center for Translational NeuroImaging at Northeastern University. Biodegradable microspheres were prepared in Dr. Yagi's laboratory at Scripps Research Institute, shipped on dry ice to Northeastern and used within a day or two or arrival. At the end of the 3-month-behavior and imaging studies, animals were sacrificed, transcardially perfused with 4% paraformaldehyde, the brains stored in cryoprotectant and shipped back to Dr. Yagi's lab for histological analysis. The imaging data are not included in this study. In a pilot study, we started with 5-month-old Long–Evans male rats weighing ca 450–500 g in accordance with the Marella publication. Two months later many of these animals exceed 600 g in body weight and outgrew the body restrainer and holders designed for awake animal imaging in the magnet. Consequently we decided to work with older but smaller, female Long–Evans rats ca 8–9 months of age and between 400 and 450 g of body weight. Over the 3 months following rotenone or vehicle treatment these animals grew to between 425 and 500 g in body weight. However, because estrogen is reported to be protective in different animal models of PD (Dluzen 1997; Leranth et al. 2000; Gao and Dluzen 2001) we ovariectomized animals 2 weeks before rotenone microsphere injection. Consequently this model examines disease progression in ovariectomized rats up to almost 1 year of age.

This study with ovariectomized aged rats was repeated three times. The first time was a pilot with four animals per vehicle and rotenone treated groups. The second time was a larger study with eight animals per vehicle and rotenone groups. The third time was another pilot of four animals per group but included a third experimental condition of rotenone plus FAAH (fatty acid amide hydrolase) inhibitor to evaluate the use of a pharmacotherapeutic to block disease progression (data not shown). In all three studies, animals were sacrificed between 10 and 12 weeks postrotenone or vehicle. The histological data for vehicle and rotenone treated animals were similar as reported for each molecular and cellular marker.

#### Test statistics

The statistical comparisons between control and rotenone treated animals for measures of motor behavior and body weights over time were done with a two-way repeated measures ANOVA followed by Bonferroni post hoc tests.

Digitized brain images were captured using a charge-coupled-device camera (XC-77; Sony, Tokyo, Japan). The density of striatal dopaminergic fibers was analyzed using Image J software (version 1.63; National Institutes of Health, Bethesda, MD). The average labeling for each area was calculated from four adjacent brain sections of the same animal at the level of the anterior commissure. Striatal images converted to gray scale were then delineated, and the intensity of staining was assessed for the entire region of four sections and subsequently averaged for each animal. Background intensities taken from the corpus callosum devoid of tyrosine hydroxylase (TH) staining were subtracted from every measurement. Statistical analyses were performed using the unpaired Student's *t*-test on StatView software (SAS institute, Middleton, MA). Data derived from the striatum and substantia nigra were expressed as mean values 6 SD. The loss of dopaminergic neurons was determined by counting the average of TH-immunoreactive neurons in the three substantia nigra pars compacta sections at high magnification (20×) under bright-field illumination (E800 Nikon microscope; Nikon Instruments, Tokyo, Japan). The cell count was performed in a masked fashion by two independent investigators. Analysis of TH-immunoreactive cells was restricted to the substantia nigra pars compacta and thus excluded the ventral tegmental area. Evaluation of staining intensity or of cell number was performed using imageJ (Rasband 1997–2012) and FIJI (Schindelin et al. 2012) software.

### Automated locomotor activity testing

Locomotor behavior was measured with eight animal activity cages (Digiscan CCDIGIJ) purchased from AccuScan Instruments, Ohio. The activity cages consisted of clear plastic acrylic (40 × 25 × 20 cm), with 16 equally spaced (2.5 cm) infrared beams across the length of the cage connected to a Digiscan Data Analyzer. Information from the analyzer was sent to a personal computer that displayed the data through a Windows-based program (DigiPro, Mukilteo, WA). The analyzer collected the beam status information and developed a dynamic picture of animal activity. The Digipro system calculates the total number of beams that are interrupted by the animal and expresses this value as locomotor counts and/or distance traveled in centimeters. Animals were tested at 14-day intervals staring on day 3 posttreatment. In the original pilot study animals were only tested on weeks 3, 5, and 7 posttreatment.

### Microspheres production

The rotenone microspheres were produced by batch according to an emulsion solvent evaporation/extraction method. The rotenone was embedded in a biodegradable polymer of poly (dl-lactide-co-glycolide) (PLGA; Sigma, St. Louis, MO). A quantity of 258 mg of rotenone was dissolved with 403 mg of PLGA (lactide:glycolide 75:25, mol wt 90,000–126,000) in 15 mL of dichloromethane. The solution was vortex at least 15 min at ambient temperature. This organic phase was poured into 300 mL of ice-cold 4% (w/v) polyvinyl alcohol (hot water soluble; Sigma). The emulsion was stirred at maximum speed for 1 h in hermetic condition. Then the seal was broken in order to evaporate the dichloromethane for 3 h at ambient temperature. The microspheres were collected by centrif-ugation and washed with distilled water. The average diameter of the beads was estimated at 35 μm. For the control batch the procedure was similar except the addition of rotenone.

### Immunohistochemistry

Cryo-embedded brains were cut on a cryostat (30 μm thickness) and collected on Superfrost slides. The slices were dried in a 42°C oven during 18 h then stored at −20°C. Immunohistochemistry experiment required the use of an antigen retrieval method. The antigen retrieval was performed in a commercial microwave oven (1600 watts). The slides were placed in a preboiled solution of 1 mM EDTA (ethylenediaminetetraacetic acid), 10 mM Tris-Cl, pH 8 and microwaved for 15 min at 20% of the maximum power of the oven (80–95°C). The solution was cooled to room temperature and the slides transferred to phosphate buffered saline (PBS) for the staining procedures.

Brain slices were washed in PBS two times during 5 min and incubated in blocking reagent (PBS pH 7.8, 10% FBS (fetal bovine serum), 0.1% triton X-100) for 2 h. The appro-priate primary antibody was applied over night at 4°C in the blocking solution (NeuN 3 μg/mL, VMAT2 2.5 μg/mL, DAT 3 μg/mL, TH 2.5 μg/mL, Ubiquitin 3 μg/mL, α-synuclein 3 μg/mL, GFAP 1.25 μg/mL, microglia CD11b 3 μg/mL). After three washes in PBS secondary antibodies were incubated at room temperature for 4 h. For fluorescent staining, the slides were mount with Vectashield (Vector Lab., Burlingame, CA). For diaminobenzidine (DAB) staining, we used biotinylated secondary antibodies revealed by the ABC kit (Vector Lab.). The slides were then counterstained with cresyl violet, dehydrated, and mounted with Permount (Fisher). Note, for DAB staining the slides were preincubated in methanol 3% hydrogen peroxide (H_2_O_2_) for 20 min before the blocking step.

The 7,8-dihydro-8-oxo-deoxyguanine (8-oxo-dG) staining was performed as previously described by Marella et al. (2007). Briefly, brain slices were treated with RNase A, then, after an incubation in 4 N HCl the acid was neutralized and the slices were blocked for immunostaining.

The determination of iron accumulation in SN was done by a method largely inspired by Nguyen-Legros et al. (1980), as a new histochemical demonstration of exogenous iron. The brain sections were immersed in a Perl's staining solution of 5% HCl, 10% potassium ferrocyanide in water at room temperature during 1 h. After three washes with ultrapure distilled water the sensitivity of the staining was increased by secondary reactions with DAB and H_2_O_2_ for 20 min. The slices were counterstained with cresyl violet, dehydrated, and mounted with Permount.

For SPECT/CT imaging animals were anesthetized during i.v. administration of ^125^I-betaCIT (0.4 mCi, 0.3 mL) and were returned to their cages after injection for the uptake period. In vivo images were acquired at 3 h postinjection using the NanoSPECT/CT® (Bioscan, Washington, DC). The NanoSPECT/CT® is a dual-modality imaging system combining a 4-headed SPECT camera with a computed tomography (CT) acquisition system on the same axis of rotation. Following anesthesia induction (4% isoflurane at 2 L/min), rats were placed on a heated bed with integrated gas anesthetic (Minerve, France). Anesthesia was administered at 2 L/min, 2% isoflurane, and the animals' body temperatures were maintained at 36–37°C for the duration of image acquisition. Each imaging time-point included three scans: a planar x-ray scout scan, a brain-focused CT scan (3 min), and a brain-focused SPECT scan (25 min). SPECT acquisitions were performed with 9-pinhole apertures (Φ = 2 mm) designed for focused rat-imaging, employing 24 angular projections and an energy window of 27 KeV ± 10%. SPECT data were reconstructed with a proprietary, raytracing-based OSEM algorithm using the HiSPECT reconstruction software platform (Scivis, Göttingen, Germany).

A quantitative calibration was performed prior to the beginning of the study using the 2-mm aperture and a dedicated rat phantom filled with a known amount of ^125^I. The quantitative calibration provides a stable scaling factor used to express reconstructed voxel values in units of radioactivity. Together, the quantitative calibration and the proprietary OSEM reconstruction algorithm facilitate absolute quantification of radioactivity measured in vivo. The quantitative capabilities of the NanoSPECT/CT® have been tested and published, showing quantification accuracy within the measurement error of a standard Dose Calibrator. As the Dose Calibrator is used to measure the input function (dose of radiotracer), the NanoSPECT/CT® is used to measure the distribution of radiotracer in vivo with equal or greater accuracy. As a result, uptake can be expressed in absolute units of radioactivity (μCi), concentration (μCi/mm^3^), or percent of injected dose (% ID).

Quantification of striatal uptake of ^125^I-betaCIT was performed using the Invivoscope postprocessing software package (Bioscan, Washington, DC). Reconstructed SPECT and CT data were loaded into the Invivoscope, manually coregistered Cylindrical volumes-of-interest (VOI) were drawn manually around each hemisphere of the dorsal striatum. Uptake and concentration values for each hemisphere were derived from these VOI's and used for analyses.

## Results

**Figure 1:**
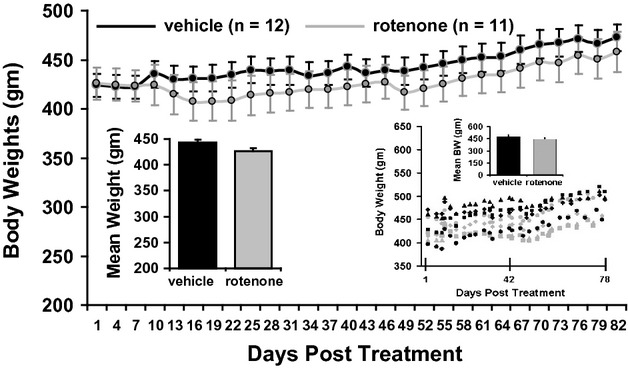
Body weights. Shown are the changes in body weights over 82 days for rats treated with vehicle (*n* = 12) of empty microspheres (black) or rats (*n* = 11) treated with rotenone filled microspheres (gray). These data are a composite of three separate studies. The lower left inset shows the mean body weight for each experimental group from the three studies. The lower inset on the right shows the individual body weights over time from the original pilot study (*n* = 4, for each group). Vertical lines denote SEM.

Shown in Figure 1 are the changes in body weights over 11 weeks for rats treated with vehicle (*n* = 12) of empty microspheres or rats (*n* = 11) treated with rotenone filled microspheres. These data are a composite of three separate studies. The lower left inset shows the mean body weight for each experimental group from the three studies. The lower inset on the right shows the individual body weights over time from the original pilot study (*n* = 4, for each group). There is no significant difference in body weights over time between the vehicle and rotenone treated animals.

**Figure 2:**
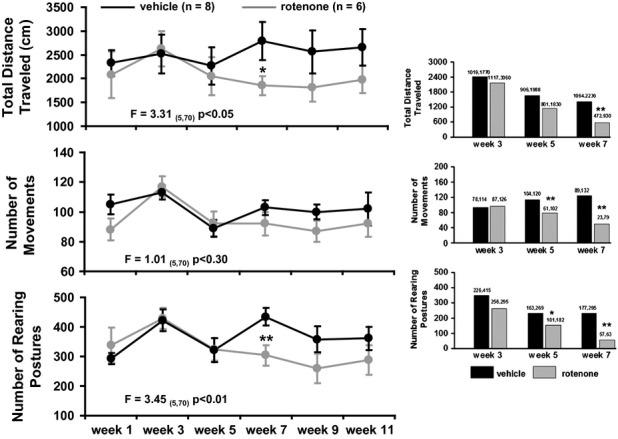
Motor behavior. Shown are the changes in different measures of motor behavior over a 11-week period as a composite of two of the three studies following motor behavior from week 1 to 11. The inset to the right shows data from the original pilots study (*n* = 4 per group) that only followed motor activity from week 3 to 7. The height of the bar graphs in this inset is the median score for each measure with the minimum and maximum shown above. A two-way repeated measures ANOVA for the 11-week study showed a significant difference between the total distance traveled and the number of rearing postures at week 7 between vehicle (*n* = 8) and rotenone (*n* = 6) treated animals. Vertical lines denote SEM. *P < 0.05, **P < 0.01.

Shown in Figure 2 are the changes in different measures of motor behavior as a composite of two of the three studies following motor behavior from week 1 to 11. The inset to the right shows data from the original pilots study (*n* = 4 per group) that only followed motor activity from week 3 to 7. The height of the bar graphs in this inset is the median score for each measure with the minimum and maximum shown above. A two-way repeated measures ANOVA for a 11-week study showed a significant difference between the total distance traveled and the number of rearing postures at week 7 between vehicle (*n* = 8) and rotenone (*n* = 6) treated animals. This would suggest a trend toward hypokinesia with rotenone treatment.

**Figure 3:**
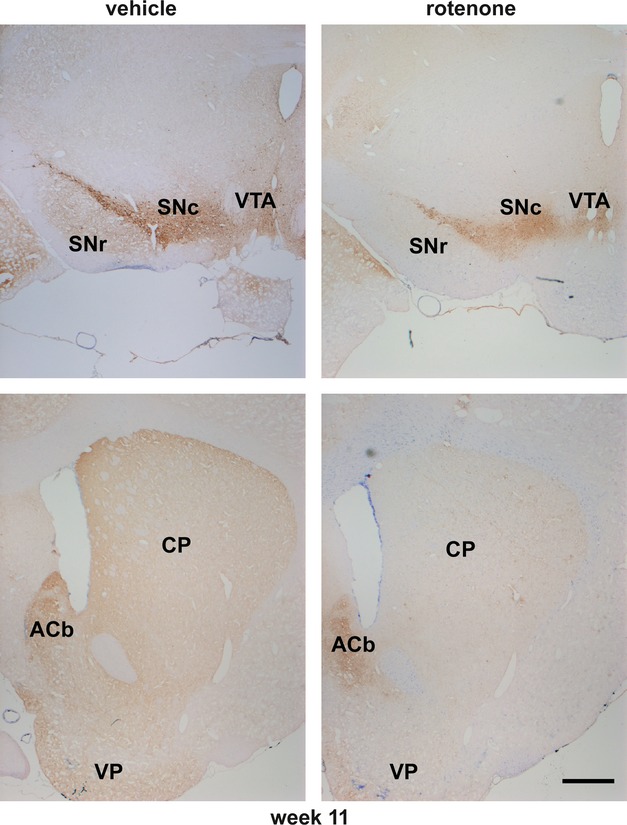
Site-specific reduction in tyrosine hydroxylase staining. Shown are representative micrographs at different magnifications of immunostaining for tyrosine hydroxylase (brown) at week 11 postvehicle or rotenone treatment. The upper two panels show immunostaining in the ventral tegmental area (VTA), substantia nigra compacta (SNC), and substantia nigra reticularis (SNR). The lower row shows immunostaining in the caudate/putamen (CP) nucleus accumbens (Acb) and ventral pallidum (VP). Scale = 500 μm.

**Figure 4:**
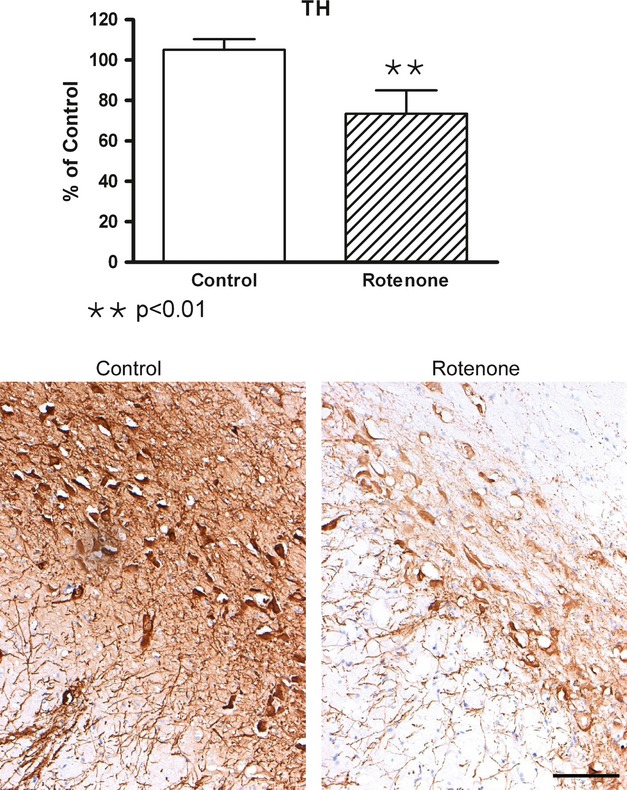
Reduction in tyrosine hydroxylase in the substantia nigra compacta. The bar graphs show the mean percent of control for vehicle (*n* = 4) and rotenone treated animals (*n* = 4) sampled from the substantia nigra compacta areas depicted in the micrographs below. Scale = 30 μm. **P ≤ 0.001 (student *t*-test/control).

**Figure 5:**
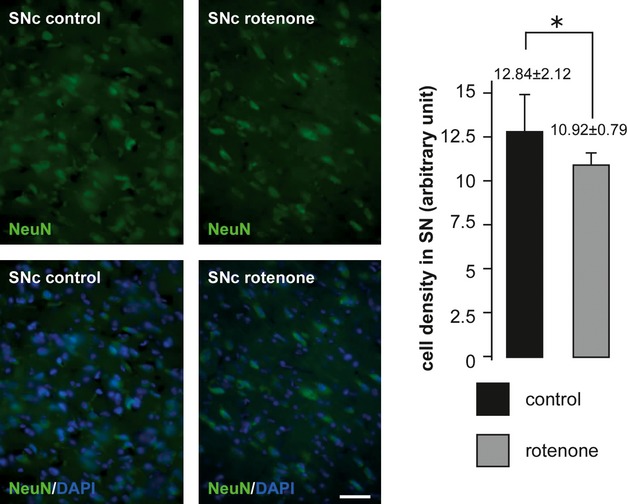
Reduction in neuron density in the substantia nigra compacta. The top panels show representative green immunofluorescence staining for the neuron's specific neuN protein in the substantia nigra compacta (SNC) 11 weeks postvehicle and rotenone treatments. The reduction in neuron density can be observed below when the neuN staining is merged with blue fluorescence DAPI (4′,6-diamidino-2-phenylindole) staining used to identify cell nuclei. The bar graphs to the right are the mean cell densities for vehicle and rotenone treatment as a composite of all three experiments. Vertical bars denote SD. Scale = 30 μm. *P ≤ 0.001 (student *t*-test/control).

Immunostaining for TH in the midbrain and striatum at week 11 postvehicle and rotenone treatment are shown in Figure 3. There is a substantial decrease in TH staining in both the substantia nigra pars compacta (SNpc) the location of DA neurons and the DA fibers in the underlying substantia nigra pars reticularis (SNpr). By week 11 there is a ca. 25% reduction in TH staining in the SNpc as shown in Figure 4. This reduction in TH staining is also accompanied by a 10–15% reduction in neuronal numbers in the SNpc as shown in Figure 5.

**Figure 6:**
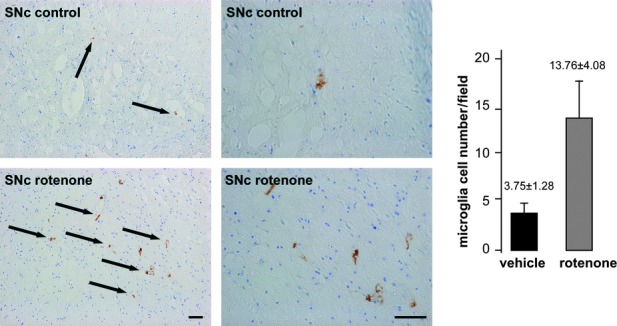
Increased numbers of activated microglia in the substantia nigra compacta. Shown are representative micrographs at different magnifications of immunostaining for activated microglia (arrows) at week 11 postvehicle or rotenone treatment. The bar graphs in the inset are microglia cell numbers for each condition (*n* = 4). Histograms are represented ±SD. Scale = 30 μm.

**Figure 7:**
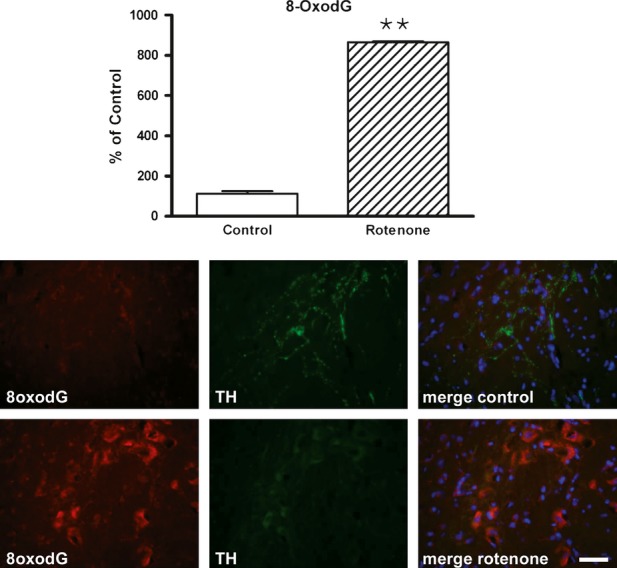
Increased levels of 8-oxo-2′-deoxyguanosine (8-oxo-dG). The first and second columns of photomicrographs from the left show representative immunofluorescence staining for 8-oxo-dG (red) and tyrosine hydroxylase (green) in the substantia nigra compacta (SNC) 11 weeks postvehicle and rotenone treatments. These images are merged with the 4',6-diamidino-2-phenylindole staining of the nucleus. The bar graphs in the inset show the level of 8-oxo-dG in rotenone animals as a percent of control (*n* = 4). Histograms are represented ±SD. Scale = 30 μm. **P ≤ 0.001 (student *t*-test/control).

**Figure 8:**
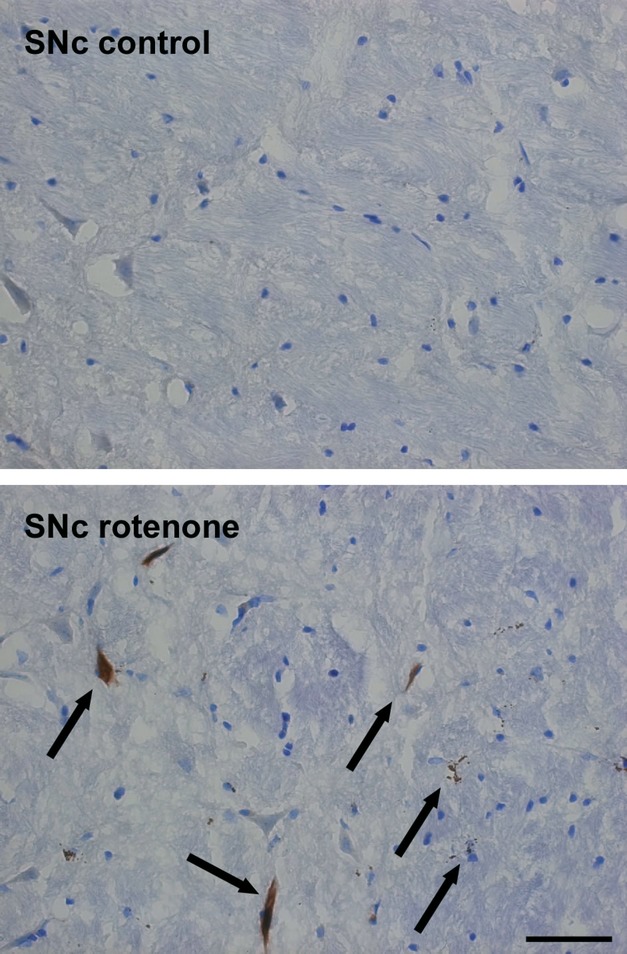
Iron accumulation. Shown is a representative example of iron accumulation in the substantia nigra compacta (lower panel, arrows) 11 weeks postrotenone treatment. Control animals showed no iron precipitates. Iron deposits such as the one shown by arrows are found in equal proportion throughout the animals treated with rotenone. Scale = 10 μm.

**Figure 9:**
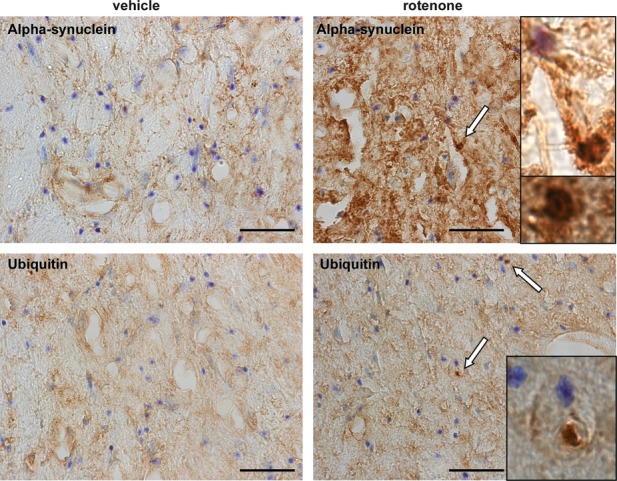
Presence of putative Lewy bodies in the substantia nigra compacta. Shown are representative photomicrographs of immunostaining for α-synuclein and ubiquitin in the substantia nigra compacta for each of the experimental conditions. Arrows point to cytoplasmic inclusions of aggregated proteins typical of Lewy bodies with a dense core of ubiquitin and “ring-shaped” staining of α-synuclein. Scale = 10 μm.

Accompanying the loss of DA neurons in the SNpc are cellular signs of neuroinflammation, oxidative stress and protein misfolding. Shown in Figure 6 are photomicrographs of immunostaining for activated microglia in rotenone treated animals as compared to control. There is a threefold increase in the number neuroinflammatory microglia in the SNpc 11 weeks postrotenone treatment. The vulnerability of the neurons in the SNpc to rotenone is further evidenced by the accumulation of 8-oxo-2′-deoxyguanosine (8-oxo-dG), an oxidized derivative of deoxyguanosine and a major product of DNA oxidation. Levels of 8-oxo-dG in the SNpc are over eightfold higher in rotenone treated animals as compared to vehicle as shown in Figure 7. There is the appearance of iron precipitate in the SNpc as shown in Figure 8. The hallmark of PD – Lewy bodies, also appear in the SNpc after rotenone treatment. The photomicrographs in Figure 9 are representative examples of α-synuclein and ubiquitin inclusions in neurons of SNpc that are typical of Lewy bodies. These protein aggregates constituting putative Lewy bodies were not observed in the dorsal striatum or other brain areas.

**Figure 10:**
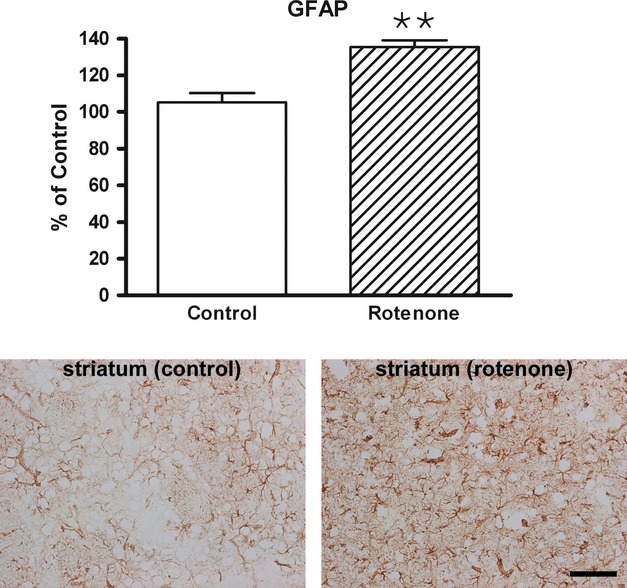
Increased levels of glial fibrillary acidic protein (GFAP) in the dorsal striatum. Shown are representative micrographs of immunostaining for activated GFAP [diaminobenzidine (DAB) revelation] at week 11 postvehicle or rotenone treatment. The bar graphs in the inset show the change in GFAP levels as a percentage of control (*n* = 4). Histograms are represented ±SD. Scale = 40 μm. **P ≤ 0.001 (student *t*-test/control).

**Figure 11:**
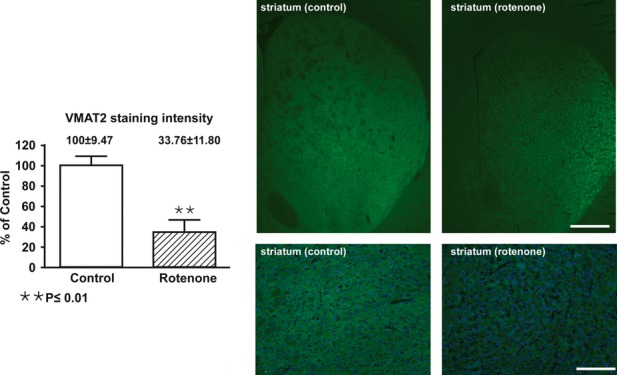
Decreased levels of vesicular monoamine transporter in the dorsal striatum. Shown in the upper panels are representative photomicrographs of immunofluorescence staining for vesicular monoamine transporter 2 (VMAT2) (green) in the dorsal striatum 11 weeks postvehicle or rotenone treatment. In the lower panels, VMAT2 staining is merged with 4',6-diamidino-2-phenylindole staining (blue). Scale = 300 μm and scale = 50 μm, respectively. The bar graphs in the inset show the levels VMAT2 in each condition (*n* = 4). Histograms are represented ±SD. **P ≤ 0.001 (student *t*-test/control).

**Figure 12:**
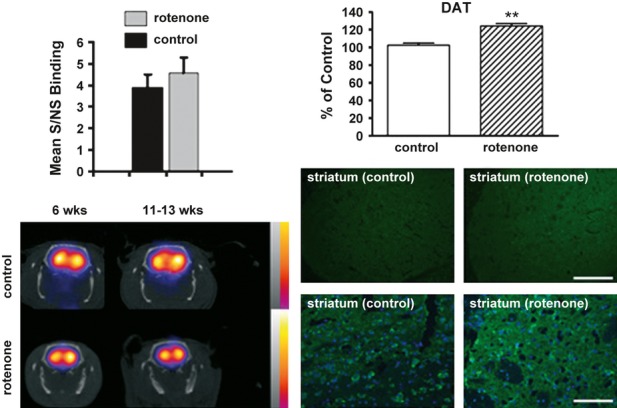
Increased levels of dopamine transporter in the dorsal striatum. Shown in the right panels are representative photomicrographs of immunofluorescence staining for dopamine transporter (DAT) (green) in the dorsal striatum 11 weeks postvehicle and rotenone treatments. These images are merged in the lower panels with 4',6-diamidino-2-phenylindole staining (blue). The bar graphs in the inset show the levels DAT as a percentage of control (*n* = 4). Scale = 300 μm and scale = 50 μm, respectively. The accompanying inset to the left shows data collected from SPECT imaging of 125I-B-CIT in the dorsal striatum of rotenone and vehicle treated animals (*n* = 6). The bar graphs show the mean specific binding as a ratio of nonspecific binding. There were no significant differences in DAT levels in the dorsal striatum as assessed by SPECT imaging. Histograms are represented ±SD. **P ≤ 0.001 (student *t*-test/control).

The immunostaining of the efferent fibers of the SNpc DA neurons projecting to the caudate/putamen (CP) of the dorsal striatum is reduced (Fig. 3). However, TH staining in fibers coming from the ventral tegmental area (VTA) projecting to the accumbens appear intact. The rotenone-mediated insult to the midbrain dopaminergic system and its projection to the dorsal striatum is further characterized by the increase in glial fibrillary acidic protein (GFAP) in the caudate/putamen as shown in Figure 10. These reactive astrocytes, which are 30% over control levels are an early sign of DA nerve terminal damage. Along with the increase in GFAP staining there is a significant reduction in the staining for VMAT2 (Fig. 11) and increase in dopamine transporter (DAT) (Fig. 12).

## Discussion

The key features that define idiopathic PD are the loss of DA neurons in the nigrostriatal pathway, the accompanying bradykinesia, and rigidity and the presence of α-synuclein inclusions in the SN (Litvan et al. 2007). Symptomatic PD is thought to occur when there is approximately 80% reduction in DA terminals in the dorsal striatum and 50% reduction in DA neurons in SN(Bernheimer et al. 1973). Characterizing the neuroanatomical localization and degree of synucleinopathy in postmortem tissue reveals PD to be a progressive, multisystem disease, affecting select populations of neurons in motor, autonomic, and limbic systems (Braak and Del Tredici 2008). The seminal work of Braak and colleagues (2004) describes the developmental stages of PD from presymptomatic synucleinopathy of olfactory and autonomic brain areas to the symptomatic involvement of the basal ganglia and cortex. Dickson and coworkers (2008) reported incidental Lewy bodies in clinically normal individuals over the age of 60 years. TH levels in the striatum were reduced in these individuals, but not to the level of PD patients. The reduction in TH in the dorsal striatum and loss of DA neurons and the presence of putative Lewy bodies in the SN in this phenotypic model recapitulating the neuropathology of Parkinson's disease is critical, and key to the characterization and relevance of this model to human PD. Consequently, synucleinopathy as evidenced in this model may be a biomarker of early loss of DA neurons that has not exceeded the threshold leading to loss of function.

The etiology of idiopathic PD is not known. It is most prevalent in aging populations around the world (Bower et al. 2000; Van Den Eeden et al. 2003). Old age along with genetic susceptibility and environmental toxins are all contributing factors to the development of PD. There is compelling data from many sources that disruption of mitochondrial respiration at complex 1 of electron transport chain in DA neurons is a contributing factor to PD (Bindoff et al. 1989; Parker et al. 1989; Schapira et al. 1989, 1990; Shoffner et al. 1991; Cardellach et al. 1993; Blin et al. 1994; Swerdlow et al. 1996; Champy et al. 2004; Perier et al. 2007). Evidence to this point began with the unfortunate, but scientifically invaluable observation where drug addicts exposed to 1-methyl-4-phenyl-1,2,3,6-tetrahydropyridine (MPTP) and its subsequent conversion to MAPP+ (1-methyl-4-phenylpyridinium), a specific inhibitor of complex 1 and a substrate for the dopamine transporter, developed signs and symptoms of idiopathic PD (Langston et al. 1983, 1999; Ballard et al. 1985). The pesticide rotenone used in this in vivo study is also a highly selective inhibitor of complex 1 and is the prototypic mitochondrial poison for in vitro studies on DA cell cultures and SN tissue slices (Gao et al. 2002; Sherer et al. 2002; Testa et al. 2005; Hsuan et al. 2006). As noted in earlier studies, rats treated with rotenone show loss of DA neurons in the SN and the confluence of mitochondrial dysfunction, synucleinopathy, microglia activation, and oxidative stress (Sherer et al. 2002, 2003a,b,c; Testa et al. 2005; Betarbet et al. 2006). Each of the components of disease progression were evidenced in this phenotypic model recapitulating the neuropathology of Parkinson's disease. While animals did not shows signs of bradykinesia, rigidity or tremors they did present with a modest reduction in motor activity that would suggest a trend toward hypokinesia.

The increased number of activated microglia in this model would be predicted if there was neuroinflam-mation in the SN. Transient activation of microglia contribute to the brain's innate immune response to acute insults by producing reactive oxygen species (ROS) and cytokines to neutralize pathogens, engulfing cellular debris, and releasing trophic factors, like brain-derived neurotrophic factor for example, to promote axonal sprouting of DA neurons (Batchelor et al. 1999). However, chronic neuroinflammation from protracted microglia activation would appear to promote a self-sustaining interaction between DA neurons and microglia that poison the microenvironment and exacerbate neurodegeneration (for reviews see Tansey et al. 2007; Whitton 2007). Proinflammatory signals from microglia, for example, TNF- α, INF- γ, IL-1β are elevated in PD as are levels of ROS associated with the increased expression of inducible nitric oxide synthase (iNOS) and nicotinamide adenine dinucleotide phosphate oxidase (Mogi et al. 1994; Hunot et al. 1996; Muller et al. 1998; Knott et al. 2000; Nagatsu et al. 2000; Gao et al. 2003a; Wu et al. 2003). These deleterious conditions persist long after the initial insult as reported in animal models of PD and humans exposed to MPTP (Gao et al. 2002; McGeer et al. 2003; Sherer et al. 2003c; Block and Hong 2005; Minghetti et al. 2005). Indeed, PD and all neurodegenerative diseases have microglia activation and neuroinflammation as part of the pathophysiology of disease progression (Vila et al. 2001; Liu and Hong 2003). Inhibition of microglia activation and production of proinflammatory factors in the SN reduce DA neurodegeneration in animal models of PD (Gao et al. 2003b; Yang et al. 2005; Zhou et al. 2007).

Oxidative stress has long been considered a major factor in the pathogenesis of PD. Evidence in support of this notion comes, in part, from the highly oxidative environment intrinsic to the SN. The SN has a high concentration of iron and DA, two reactive species prone to oxidative modification (Jenner 1998; Greenamyre et al. 1999). The oxidant hydrogen peroxide is a normal by-product of the deamination of DA by monoamine oxidase (Gotz et al. 1994). This high oxidative environment promotes deposition of ubiquitin and alpha synuclein inclusion or putative Lewy bodies in the cytoplasm of DA neurons (Spillantini et al. 1997). The naturally occurring antioxidant glutathione is lower in the SN of PD (Bharath et al. 2002) Adding to the vulnerability of the SN to oxidative stress is its high density of microglia as compared to other brain areas (Kim et al. 2000). As noted above, microglia activation and release of proinflammatory cytokine promotes oxidative stress. TNF- α, INF- γ, IL-1β can all activate iNOS contributing to the formation of the highly active ROS, nitric oxide (Hunot et al. 1996; Delgado 2003). Postmortem SN samples from PD patients show elevated numbers of microglia coexpressing iNOS as compared to controls (Hunot et al. 1996; Knott et al. 2000). Thus, activated microglia and their production of ROS is thought to be the major source of oxidative stress contributing to the death of DA neurons in PD (Jenner 1998; Koutsilieri et al. 2002) and the accumulation of ferrous ions, decreased glutathione (Bharath et al. 2002). Indeed, iron deposition in the SN is another hallmark of PD (Hirsch et al. 1991; Sofic et al. 1991; Song et al. 2007) as is increased DNA damage due to oxidation of guanine and the formation of 8-oxo-dG (Fleming et al. 1994; Alam et al. 1997; Zhang et al. 1999; Kikuchi et al. 2002). Again, both measures of oxidative stress are present in this model of PD.

As noted above, the susceptibility of DA to oxidative modification can contribute to the toxic environment of SN. The metabolism and auto-oxidation of DA in the cytosol of SN neurons is safeguarded, in part, by the sequestration of DA in synaptic vesicles. This function is carried out by VMAT2 (for review see Taylor et al. 2011). The activity of VMAT2, in addition, to regulating synaptic neurotransmission, confers a level of protection to cellular damage in DA nerve terminals. Loss of VMAT2 function might be expected to be one risk factor contributing to the pathophysiology of PD. Levels of VMAT2 are reduced in the striatum of PD brain samples (Miller et al. 1999) and in positron emission tomography (PET) studies on PD patients (Kilbourn et al. 1993; Frey et al. 1996; Lee et al. 2000; Martin et al. 2008; Okamura et al. 2010). VMAT2 levels correlate with the severity of Parkinsonism; hence, PET imaging of VMAT2 offers a sensitive in vivo method for detecting the early loss of DA nerve terminals in the striatum and may serve as a biomarker of presymptomatic PD. The significant decrease in VMAT2 immunostaining in this rotenone microsphere model PD supports this notion

The most intriguing aspect of this model of PD was the modest but significant increase in DAT, the dopamine transporter. DAT is widely used as a molecular biomarker to assess the integrity of presynaptic DA nerve terminals in the caudate/putamen (for review see Brooks 2010). Loss of DA terminals in the caudate/putamen is associated with a loss of DAT binding. There is a decline in DAT binding that defines a threshold for early Parkinsonism (Guttman et al. 1997) making it possible to follow disease progression in PD patients (Nurmi et al. 2000; Marek et al. 2001; Winogrodzka et al. 2001). DAT binding aids in the early diagnosis of PD from other motor disorders helping to detect patients at baseline who after follow-up months or years later show no change in status (Jennings et al. 2004; Marshall et al. 2009). In addition, as compensation for the decrease in DA terminals, there is down-regulation of DAT protein helping promote higher sustained levels of DA in the synaptic cleft (Lee et al. 2000).

Could there be a compensatory increase in DAT in presymptomatic PD? Compensation could occur by sprouting new terminals or by increasing DAT protein expression in surviving nerve terminals. Several studies in rodents have reported selective lesions to the DA innervation of the dorsal striatum are accompanied by sprouting of healthy neurons surrounding the site of injury (Dravid et al. 1984; Blanchard et al. 1996; Batchelor et al. 1999; Bezard et al. 2000). However, this is unlikely in this rotenone microsphere model as TH staining, an indirect measure of DA terminals in the dorsal striatum is significantly reduced. It is more plausible to assume an increase in DAT protein, a possibility raised by Bellucci and coworkers (2011) working with 12-month-old SYN120 transgenic mice expressing a truncated human α-synuclein. These mice show an age-dependent increase in α-synuclein deposition in the soma and dendrites of DA neurons of the SN and increased numbers of activated microglia in the surrounding tissue (Tofaris et al. 2006). While there is no decrease in the number of TH positive neurons in the substantia nigra there is a 30% decrease in DA levels and reduced DA release in the dorsal striatum (Tofaris et al. 2006; Garcia-Reitbock et al. 2010). The behavioral phenotype presents with reduced locomotion at 12–18 months of age as compared to age-matched controls, but there are no signs and symptoms of Parkinsonism (Tofaris et al. 2006; Bellucci et al. 2011). These mice show complexes of DAT/α-synuclein clustering in the cytosol of DA fibers in the striatum that accumulate with age as compared to controls (Bellucci et al. 2011). These changes are accompanied by a significant increase of DAT protein (Bellucci et al. 2011). There is a direct protein–protein interaction between α-synuclein and DAT that is thought to function as a negative regulator of DA neurotransmission (Wersinger and Sidhu 2003; Eriksen et al. 2010; Swant et al. 2011). The gradual and subthreshold loss of DA function in the striatum of these SYN120 transgenic mice, together with the accumulation of α-synuclein aggregates, increase in DAT levels, and tissue neuroinflammation, without motor signs of PD have many similarities with the rotenone microsphere model described here. Interestingly, the two models exploit different risk factors – mitochondrial stress and over expression of α-synuclein – to produce a similar neurobiological and behavioral phenotype. The convergence of these two separate risk factors may help shed light on the time and age dependent molecular and cellular mechanisms contributing to Parkinsonism.

## Summary

This study describes the methodology and characterization of a phenotypic model recapitulating the neuropathology of PD in aged ovariectomized rats using the mitochondrial toxin rotenone, administered in biodegradable microspheres. Animals appear healthy but do display a modest decrease in motor behavior and trend toward hypokinesia. The motor signs, for example, tremor, rigidity, bradykinesia of Parkinsonism are absent. Yet, there is a significant loss of dopaminergic innervation to the dorsal striatum and putative DA neurons in the substantia nigra compacta. These changes are accompanied by an increase in activated microglia, iron precipitates and 8-oxo-2′-deoxyguanosine, all evidence of enhanced neuroinflammation and oxidative stress in the area of substantia nigra compacta. The increase in reactive astrocytes in the dorsal striatum together with diminished tyrosine hydroxylase levels are evidence of damage to DA nerve terminals. Levels of VMAT2 are significantly reduced in the dorsal striatum; however, there is an unexpected increase in dopamine transporter levels. In the addition to all these molecular and cellular biomarkers of disease progression, there is the appearance of putative Lewy bodies, the cardinal sign of PD. This model would appear to recapitulate the many aspects of disease progression in PD and other neurodegenerative diseases. As such, it offers an opportunity to investigate new intervention strategies could arrest the loss of DA neurons and potentially restore normal dopaminergic neurotransmission.
